# Thyroid Eye Disease With Divergence Insufficiency Causing Recurrent Falls in an Elderly Patient

**DOI:** 10.7759/cureus.21695

**Published:** 2022-01-28

**Authors:** Wei Chen Lai, Nguyen S Nguyen, Azam Husain, Sachin D Rajpal, Miriam B Michael

**Affiliations:** 1 Ophthalmology, University of Maryland School of Medicine, Baltimore, USA; 2 Ophthalmology, Howard University College of Medicine, Washington, DC, USA; 3 Internal Medicine, Howard University, Washington, DC, USA; 4 Internal Medicine, University of Maryland, Baltimore, USA

**Keywords:** prism prescription, diplopia, recurrent falls, divergence insufficiency, thyroid eye disease

## Abstract

Recurrent falls are a common cause of morbidity in the elderly population, as more than one-third of individuals aged 65 years or older experience falls each year. Falls remain a multifactorial phenomenon that can potentially result in devastating debilitation and hence require proper medical attention and management. In an elderly patient presenting with recurrent falls, the workup for differential diagnoses remains wide with various causes such as postural hypotension, syncope, seizures, arrhythmia, medication-induced, and cognitive impairment. In this report, we discuss an interesting case of recurrent falls in an elderly woman with hyperthyroidism who was repeatedly found to have unremarkable lab results and negative imaging studies. She was later diagnosed with divergence insufficiency with intermittent esotropia secondary to thyroid ophthalmopathy, which was the cause of her underlying horizontal diplopia contributing to her falls. This can cause blurry vision at further distances, which is observed especially in individuals older than 50 years. Treatment typically involves prism therapy, surgery in refractory patients, and, currently, novel therapy using teprotumumab infusion. The patient was referred to see a strabismus specialist for prism prescription and possible surgical intervention. In elderly patients with a history of recurrent falls, a comprehensive visual exam should be strongly considered, especially for individuals with repeated negative workups, to prevent further testing or procedures.

## Introduction

In the United States, falls among adults over the age of 65 years are the leading cause of visits to emergency departments (EDs). Factors that increase the likelihood of falls in the elderly include advanced age, medications, as well as cognitive and sensory impairment. Of note, 33% of community-dwelling elders and 60% of nursing-home residents suffer from falls annually. A higher mortality rate following a fall is associated with advanced age among both sexes irrespective of racial and ethnic groups [1]. The crucial consequences of falls include fear of ambulating and falling; decreased mobility; and serious, at times fatal, injuries. Fractures are the most common injuries that require hospitalization [2]; among elderly men, these include fractures of the hip, ribs, spine, humerus, and pelvis, while elderly women often suffer from fractures of the hip, humerus, wrist, pelvis, and ankle [3]. The most common sources contributing to falls in the elderly include environmental risks, gait and balance disturbances, arthritis-related pain and weakness, medications or intoxication, acute illness, confusion and cognitive deficit, postural hypotension, visual disorder, neurological disorders, syncope, drop attacks, and epilepsy [4].

## Case presentation

An 84-year-old female with a past medical history of hypertension, hyperthyroidism due to Graves' disease (which had resolved), asthma, and type 2 diabetes mellitus presented to the ED with dizziness following an episode of syncope witnessed by EMS. At the initial presentation, she reported occasionally bumping into objects, tripping, and dizziness associated with standing up. The patient also endorsed photophobia and reddened eyes, as well as the newly developed double vision that is made worse with left or right lateral gaze. She could not specify when this double vision had begun. Her double vision worsened when standing up as she reported “seeing double” at a distance. Over the last several months, she had noted decreased appetite and oral intake accompanied by fatigue, weakness, and increased urinary frequency. Her symptoms had prompted her to present to the ED multiple times over the past several months with similar concerns, which had concluded with negative workups. She denied any chest pain, shortness of breath, nausea, vomiting, fevers, chills, dysuria, hematuria, blurry vision while wearing glasses, or eye pain.

In the ED, the patient’s blood pressure was initially 119/77 mmHg but increased to 160/73 mmHg without specific intervention. Complete blood count (CBC), comprehensive metabolic panel (CMP), thyroid-stimulating hormone (TSH), and serum glucose levels were obtained with overall unremarkable results (summarized in Table 1). The patient was admitted and a complete workup was performed. Urinalysis and toxicology panels were unremarkable. Chest X-ray and CT head and brain without contrast were obtained and both did not show any acute abnormalities. Her orthostatic vitals were stable, rapid plasma reagin (RPR) was nonreactive, and vitamin B12 levels were within normal limits. She had appropriate cardiac function with echography showing an ejection fraction >60%, mild acute regurgitation, and trace tricuspid regurgitation. Physical therapy and occupational therapy also evaluated the patient and recommended a rolling walker and shower chair as precautions. 

**Table 1:** Summary of laboratory results BUN: blood urea nitrogen; TSH: thyroid-stimulating hormone; RPR: rapid plasma reagin

Laboratory investigation	Result	Unit	Reference range
White blood cells	3.5	K/mcL	4.5–11.0
Hemoglobin	13.1	g/dL	11.9–15.7
Platelets	262	K/mcL	153–367
Sodium	139	mmol/L	137–145
Calcium	9.6	mg/dL	8.4–10.2
BUN	13	mg/dL	7–17
Creatinine	0.91	mg/dL	0.7–1.50
Albumin	4.8	g/dL	3.5–5.0
Glucose	125	mg/dL	74–106
TSH	1.10	mIU/L	0.47–4.68
Vitamin B12	325	pg/mL	239–931
RPR	Nonreactive		

**Figure 1:**
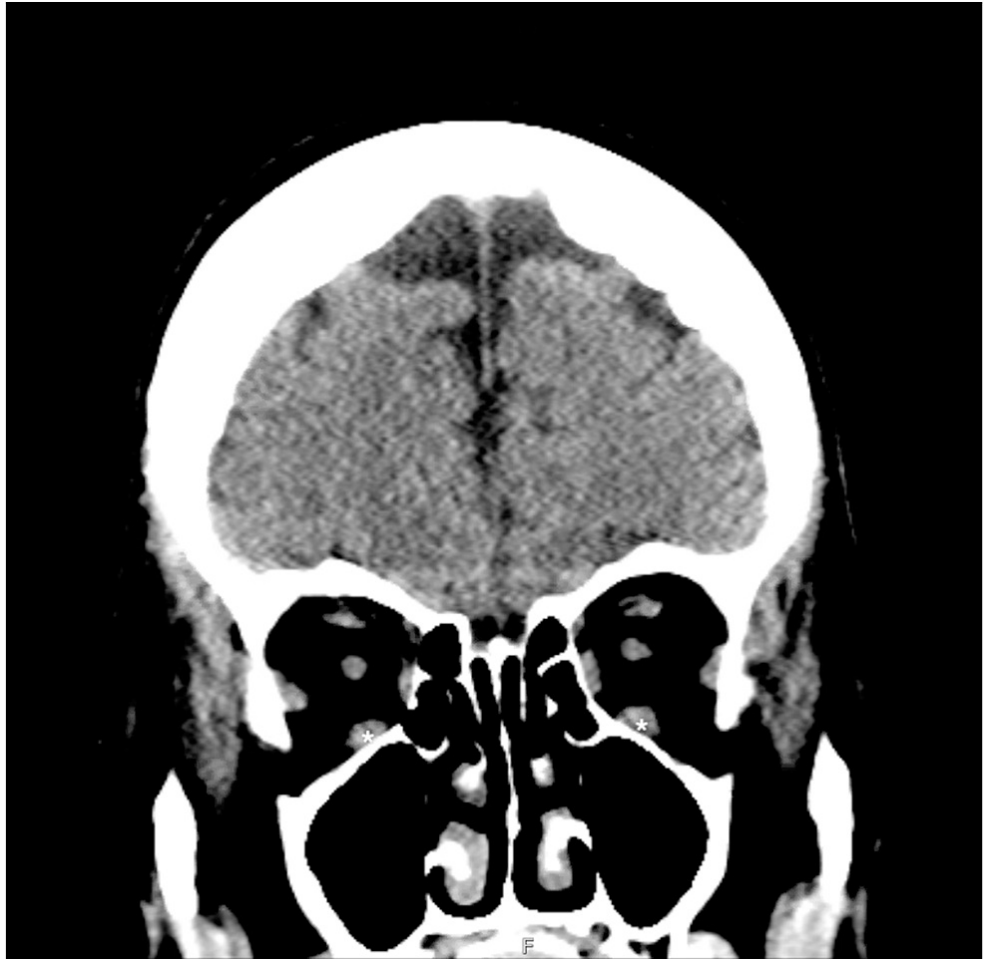
Selected CT coronal image of the head without contrast demonstrating mild enlargement of the inferior recti muscles (asterisks) CT: computed tomography

**Figure 2:**
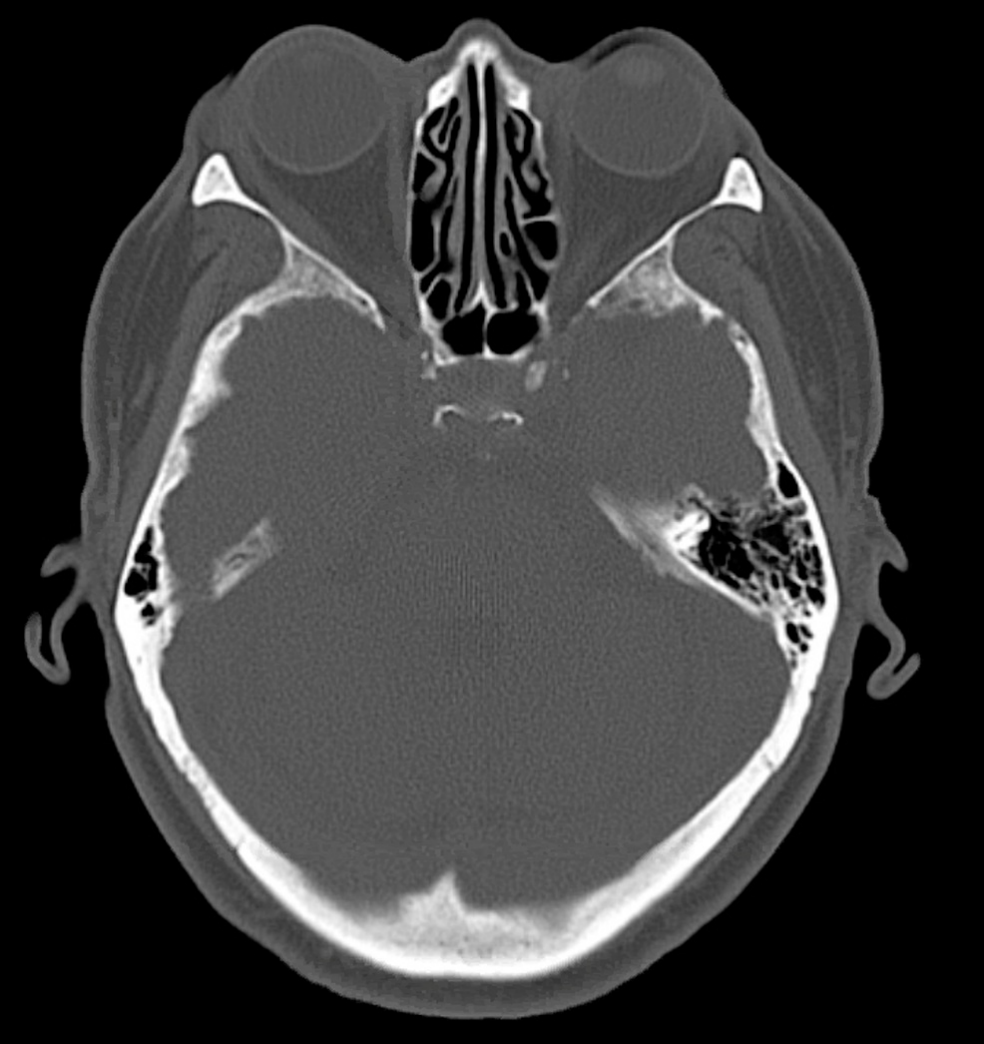
Selected CT axial image of the head without contrast demonstrating sparing of lateral recti and medial recti muscle enlargement CT: computed tomography

Given her clinical and laboratory findings, the patient was subsequently evaluated by the ophthalmology department. Further history was obtained, which revealed that she had been worked up in the past and diagnosed with thyroid eye disease (TED) due to Graves' disease. Diabetes and other autoimmune causes such as myasthenia gravis had been ruled out. At that time, the patient had received Fresnel prism lenses (a year ago), and when wearing them, her diplopia tended to resolve. However, the patient reported having many pairs of glasses and she sometimes forgot which ones were “for the double vision.” She was not wearing her Fresnel prism lenses during this admission. She was noted to present with slight lagophthalmos with eyelid retraction and no signs of exophthalmos in both eyes on physical exam. A comprehensive eye exam was performed in the ophthalmology clinic, which confirmed a diagnosis of divergence insufficiency with intermittent esotropia and thyroid ophthalmopathy. Her previous CT scan of head and orbit had not shown any signs of exophthalmos but had been suggestive of increased inferior rectus muscle belly (Figure 1). It had also demonstrated sparing of lateral recti and medial recti muscle enlargement (Figure 2). The patient was advised to return for an MRI during future clinic visits. When comparing her visit this time to her last visit a year ago, her refraction measurements had slightly changed, and she also had new cataracts in both eyes that necessitated phacoemulsification. The ophthalmology team recommended a return to the clinic to see a strabismus specialist for repeat measurements and a possible new Fresnel lens prescription.

## Discussion

When presenting to the ED, elderly patients who suffer falls undergo an extensive evaluation to determine and implement treatment for the underlying etiology. Early detection and treatment can contribute to a significant reduction in morbidity and mortality among fall patients and the subsequent substantial improvement in quality of life for both the patient and their caretakers [1]. Evaluation of a patient who presents following a fall includes a focused history, review of the system, medication use, followed by physical examination and tests to evaluate the patient’s postural control and overall physical function [5]. Following a thorough history and physical exam, laboratory workups are especially helpful in determining underlying conditions that had led to the fall. These include basic metabolic panel, renal function tests, serum medication levels, serum cardiac enzymes (if myocardial infarction is considered), CBC, thyroid function tests, and electrocardiography [6,7,8]. Imaging studies are performed to assist in the identification of predisposing conditions to the fall in addition to the consequences of the fall. For instance, a CT scan of the brain to rule out hydrocephalus or tumors and assess for signs of head injury with possible occult subdural hematoma is performed [9].

Visual disturbances, including visual acuity, contrast sensitivity, and stereo acuity, have been documented as being correlated with an increased risk of falls among the elderly [10,11,12]. However, reports on the relationship between diplopia, particularly that stemming from esotropia, and falls are scarce. Diplopia, or seeing double, is a common visual complaint among elderly patients. Its etiology can be ocular or neurological, including monocular diplopia, binocular diplopia, cranial nerve palsies, convergence insufficiency, divergence insufficiency, thyroid ophthalmopathy, myasthenia gravis, intranuclear ophthalmoplegia, postoperative diplopia, and sagging eye syndrome [13,14]. In assessing a patient’s complaint of diplopia, a physician must exclude other symptoms that can misconceive the patient, for instance, image distortion, visual field defects, afterimages, and hemianopia [14].

This report discussed a case of recurrent falls in an elderly woman with repeated negative laboratory workup, who was later diagnosed with divergence insufficiency with intermittent esotropia secondary to thyroid ophthalmopathy. Divergence insufficiency, also known as “age-related distance esotropia,” commonly manifests in elderly patients and presents as horizontal diplopia at distance. Common causes include high myopia with a long anterior-posterior axis, orbital connective tissue degeneration [15], and sixth nerve palsy associated with an underlying neurological disorder. TED leading to double vision is most often diagnosed with the help of CT or MRI, which can demonstrate extraocular muscle (EOM) involvement and enlargement. Other etiologies of strabismus, such as sagging eye syndrome (SES), can also be demonstrated on MRI. SES is a spectrum disorder and can cause binocular diplopia due to lateral rectus and superior rectus band degeneration causing lateral muscle pulley displacement and small-angle horizontal and vertical strabismus seen most commonly in elderly patients. These signs were not seen on CT orbit (Figure 2). Given our patient’s clinical presentation, CT orbit, and complete ophthalmic exam, her signs and symptoms proved sufficient for a diagnosis of divergence insufficiency with esotropia.

Divergence insufficiency is often managed conservatively with base-out prism corrective lenses, which may, however, cause diplopia if worn at near. For patients who do not show improvement or are intolerant to prism glasses or in whom misalignment is stable and long-standing, orthoptic exercises and surgery can be considered. Many patients have reported resolution of divergence insufficiency without treatment [16]. In a recent randomized clinical trial among patients with active moderate to severe ophthalmopathy, the use of immunotherapy with teprotumumab demonstrated reduced proptosis and improvement in quality of life in patients with active thyroid-associated ophthalmopathy. Although this would not be used in this patient who did not have active disease, this study shows promising advancement in treatment options in the near future [15].

Thyroid ophthalmopathy is a sequela of thyroid diseases and can affect all EOMs in varying degrees. The pathophysiology of TED lies in the increased size of the EOMs and retro-ocular connective tissue from increased fibroblast proliferation, an inflammatory response, and deposition of glycosaminoglycans. This can lead to fluid accumulation intramuscularly and eventual fibrosis. Involvement of the medial rectus (or pseudo-abducens nerve palsy) can cause abduction restriction [17]. Medial rectus muscle restriction with Graves’ disease may cause comitant esotropia at distance [18], as tight adduction from near-target convergence leads to a shortening of the medial rectus muscle and reduced ability to maintain the orthographic position at distance [19]. The screening should consist of the assessment of EOMs in the office, forced duction test to assess the direction of restriction, orbital imaging (CT or MRI scan) to assess the enlargement of EOM, thyroid function test, and autoimmune markers (thyroid-stimulating immunoglobulin, thyroglobulin antibody, and thyroid peroxidase antibody). Thyroid ophthalmopathy can typically be managed with thyroid dysfunction therapy, corticosteroid therapy, ocular occlusion for diplopia, surgery, radiotherapy, or immunotherapy [20].

## Conclusions

This report discussed the case of a patient who underwent multiple unremarkable lab workups and imaging tests that could have been avoided with an early eye examination. Her history of hyperthyroidism prompted further investigation for TED, as her diagnosis of divergence insufficiency esotropia resulting in diplopia ultimately explained the root cause of her falls. Therefore, we strongly encourage a timely and comprehensive visual history and ophthalmological examination in patients diagnosed with recurrent falls, especially if they have had multiple similar admissions with negative workups.
